# Pro-inflammatory LPS drives production and release of the chemokine MCP-1 in human coronary artery smooth muscle cells

**DOI:** 10.1007/s11010-026-05532-y

**Published:** 2026-04-11

**Authors:** Elisabeth Bankell, Olof Gidlöf, Bengt-Olof Nilsson

**Affiliations:** 1 Department of Experimental Medical Science, Lund University, BMC D12, SE-22184 Lund, Sweden; 2 Department of Cardiology, Clinical Sciences, Lund University, Skåne University Hospital, BMC D12, SE-22184 Lund, Sweden

**Keywords:** Atherosclerosis, Chemoattractant, Inflammation, Protein synthesis, Secretion, Vascular smooth muscle cells

## Abstract

The chemokine monocyte chemoattractant protein-1 (MCP-1) plays an important role as chemoattractant for monocytes in atherosclerosis. It is established that MCP-1 is produced by vascular smooth muscle cells, but the underlying mechanisms for its release are not identified. Here, we investigate production and secretion of MCP-1 in primary human coronary artery smooth muscle cells. We demonstrate that the cells express MCP-1 using RT-qPCR, immunocytochemistry and ELISA, and the ELISA analysis shows that they contain high basal levels of MCP-1 compared to human THP-1 monocytes included as positive control representing an immune cell. Immunocytochemistry discloses co-staining for MCP-1 and the ER marker calreticulin, suggesting that they may co-exist. The cellular production of MCP-1 is stimulated by the bacterial endotoxin LPS demonstrated both on mRNA and protein levels. Conditioned medium contains higher amounts of MCP-1 than fresh medium, and pro-inflammatory LPS and TNF-α stimulate release of MCP-1 from the cells. LPS does not enhance the secretion of MCP-1 at an early time point (60 min) neither in the presence nor in the absence of protein synthesis inhibition with cycloheximide, and it has no effect on intracellular [Ca^2+^] within 0–60 min, suggesting that LPS has no direct effect on the secretory process of MCP-1. We conclude that human coronary artery smooth muscle cells contain high levels of MCP-1, and that pro-inflammatory stimulus triggers secretion of this important chemokine indirectly via activation of MCP-1 production.

## Introduction

The chemokine monocyte chemoattractant protein-1 (MCP-1 or *CCL2*) is produced by many different cell types including vascular smooth muscle cells, and it acts as a potent chemoattractant for monocytes [1–3]. MCP-1 is supposed to be stored in vesicles and then secreted, but little is known about the mechanisms behind its release. When MCP-1 has been secreted, it binds to MCP-1 receptors on monocytes, and the activated receptor triggers them to migrate towards the source of MCP-1 [1]. The MCP-1 protein has a rapid turnover, and interestingly the complement system protein globular C1q receptor, also named p33, binds and stabilizes MCP-1 and thus protects it from degradation [4]. Although, it has been reported that MCP-1 is produced by vascular smooth muscle cells and released from these cells, very little is known about the exact mechanisms behind the release of MCP-1 [5].

MCP-1 receptor deficient mice show reduced arterial accumulation of lipids and amelioration of atherosclerosis, implicating that MCP-1-induced recruitment of monocytes to the vascular wall is involved in the pathogenesis of atherosclerosis [6, 7]. In humans, the lack of MCP-1 is considered to reduce vascular inflammation and to stabilize atherosclerotic plaques, thereby lowering the risk for stroke and myocardial infarction [8]. Furthermore, the concentration of MCP-1 is increased in plasma of patients with acute coronary syndromes compared to plasma of healthy volunteers, implying that MCP-1 may represent a biomarker for coronary heart disease [9]. MCP-1 gene polymorphisms are associated with increased carotid intima-media thickness providing further evidence that MCP-1 is important in human vascular pathology [10]. Atherosclerosis is an inflammatory disease, and MCP-1 is considered to be an important player in the disease process [11–13]. Therefore, it is of high in vivo relevance to study underlying mechanisms behind production of MCP-1 in vascular smooth muscle cells and its secretion from these cells.

Here, we investigate mechanisms and pathways responsible for the release of MCP-1 in primary human coronary artery smooth muscle cells (hCASMCs) stimulated with the pro-inflammatory bacterial endotoxin and TLR4 agonist LPS. We conclude that hCASMCs harbour high amounts of MCP-1, and we demonstrate for the first time that LPS triggers secretion of the chemokine from these cells, secondary to LPS-induced stimulation of MCP-1 production.

## Materials and methods

### Cells and cell culture

Primary human coronary artery smooth muscle cells (hCASMCs) were purchased from Thermo Fisher Scientific (cat. no. C0175C) and cultured in Human Vascular Smooth Muscle Cell Basal Medium (Gibco, Thermo Fisher Scientific, cat. no. M231500) supplemented with 5% (v/v) smooth muscle growth supplement (Thermo Fisher Scientific, cat. no. S00725) and antibiotics (penicillin 50 U/ml and streptomycin 50 µg/ml, Biochrom, cat. no. A2212). The hCASMCs were used for experiments in passages 4 to 9. The human monocyte cell line THP-1 cells were from ATCC (cat. no. TIB-202) and cultured in RPMI 1640 Medium, GlutaMAX (Gibco, Thermo Fisher Scientific, cat. no. 61870036) supplemented with antibiotics (penicillin 50 U/ml and streptomycin 50 µg/ml, Biochrom, cat. no. A2212) and 10% fetal bovine serum (Biowest, cat. no. S1810). The THP-1 cells were included as positive control. The cells were cultured in a cell/tissue incubator at 37 °C with 5% CO_2_ in air. Medium was exchanged every second day, and cells were reseeded upon reaching confluence. Cells were inspected in a phase-contrast microscope (Olympus CKX41) and counted with an automatic cell counter (LUNA, Logos Biosystems). Experiments were conducted in 6-well plates with 250 000 hCASMCs or 1 million THP-1 cells seeded in each well at the start of the experiments. For measurement of intracellular [Ca^2+^], cells (20 000 per well) were seeded in 96-well plates. Conditioned medium was always replaced with fresh culture medium, and cells were allowed to equilibrate for 20 min at 37 °C in the cell/tissue incubator before the experiments were started. Cell viability was determined with the trypan blue exclusion test. Briefly, cell suspensions were incubated (1:1) with trypan blue solution (0.4%, Sigma-Aldrich, cat. no. T8154), and cell viability determined in the LUNA automated cell counter (Logos Biosystems).

### Immunocytochemistry

Cells were seeded on glass coverslips (30 000 per coverslip) and allowed to adhere for 24 h in the incubator. The cells were carefully washed with PBS (37 °C) and fixed with 4% formaldehyde for 10 min. After fixation, cells were washed with PBS before they were permeabilized using Triton X-100 (0.1%) for 10 min and non-specific binding sites were blocked with BSA (2%) for 1 h at 4 °C. Cells were incubated with a rabbit polyclonal MCP-1 antibody (Abcam, cat. no. Ab9669) at dilution 1:500 overnight at 4 °C. For co-staining experiments, the same cells were incubated with a mouse monoclonal MCP-1 antibody (1:200, R&D Systems, cat. no. MAB679) and a rabbit monoclonal calreticulin antibody (1:200, Cell Signaling, cat. no. 12238) overnight at 4 °C. After washing in PBS, cells were incubated with a goat anti-rabbit Alexa Fluor 488 secondary antibody (Thermo Fisher Scientific, cat. no. A-11008, 1:500), a goat anti-mouse Alexa Fluor 488 secondary antibody (Invitrogen, cat. no. A32723, 1:500) or a goat anti-rabbit Alexa Fluor 555 secondary antibody (Invitrogen, cat. no. A21428, 1:500) for 1 h. The cells were washed with PBS before the coverslips were mounted on glass slides using the Fluoroshield with DAPI (nuclear marker) mounting medium (Sigma-Aldrich, cat. no. F6057). MCP-1 and calreticulin immunoreactivities and DAPI fluorescence were analyzed with a fluorescence microscope (Olympus BX60). Overlay of MCP-1 and calreticulin immunoreactivities and DAPI to assess co-existence was analyzed with ImageJ Fiji (NIH). No staining was observed after omission of the primary MCP-1 and calreticulin antibodies representing negative controls.

### ELISA

Cells were harvested in ice-cold PBS and cell lysates prepared by sonication (2 × 10 s). Cell lysates were centrifuged (1700 ***g***, 4 °C, 5 min) and ELISA performed on the supernatants. Cell medium was collected and gently centrifuged (82 ***g***, 20 °C, 5 min) to remove cells and cell debris. MCP-1 levels in cell lysates and cell culture media were determined by ELISA according to instructions by the manufacturer (R&D, cat. no. DPC00). Total protein was determined with the DC Protein Assay (Bio-Rad, cat. no. 5000113 reagent A, cat. no. 5000114 reagent B, cat. no. 5000115 reagent S), and each sample was analyzed in duplicate.

### Dot blot analysis

MCP-1 protein levels in culture media were assessed by dot blot. Media (1 µl) were spotted on nitrocellulose membranes (Bio-Rad, cat. no. 1620168) using PBS as blank. Membranes were blocked in tris-buffered saline with 1% casein (Bio-Rad, cat. no. 1610782) for 1 h and incubated with a rabbit polyclonal MCP-1 antibody from Abcam (cat. no. Ab9669, 1:2000) for 24–48 h at 4 °C. Membranes were carefully washed in tris-buffered saline with 0.1% tween 20 and incubated with horseradish peroxidase-conjugated anti-rabbit IgG (Cell Signaling, cat. no. 7074) at a dilution 1:5000 for 2 h. After washing, membranes were incubated with West Femto chemiluminescence reagent from Thermo Fisher Scientific (cat. no. 34096), and MCP-1 immunoreactivity visualized and analyzed with a LI-COR Odyssey instrument (LI-COR Biosciences).

### Quantitative real-time RT-PCR measurements

Cells were washed in ice-cold PBS and lysed with RLT lysis buffer (Qiagen, cat.no. 1015762). RNA was extracted and purified with the RNeasy kit and the QIAcube system (Qiagen, cat. no. 74104). RNA amounts and quality were assessed in the NanoDrop 2000 C machine (Thermo Fisher Scientific). Gene expression was analyzed using one-step qPCR with QuantiNova SYBR Green PCR kit (Qiagen, cat.no. 208156) in a Step One Plus cycler (Applied Biosystems). Each sample was analyzed in duplicates (two technical replicates), and we used the mean Ct value to determine gene expression in one sample with ≥ 3 biological replicates in each group. The MCP-1 (*CCL2*) transcript levels were analyzed with the delta-delta Ct method applying 18 S (18 S ribosomal RNA) as reference gene [14]. The MCP-1 (Hs_CCL2_1_SG, cat. no. QT00212730) and 18 S (Hs_RRN18S_1_SG, cat. no. QT00199367) QuantiTect primers were from Qiagen.

### Measurements of intracellular [Ca^2+^]

Cells were seeded in 96-well plates with dark bottom (Corning, cat. no. 3916) and allowed to attach and equilibrate for 24 h. They were loaded with the Ca^2+^-indicator Fluo-4 AM (2.5 µM, Invitrogen, cat. no. F14201) for 60 min at 37 °C in PBS with 0.05% (v/v) Pluronic F-127 (Molecular Probes, cat. no. P1572) and then washed two times in PBS. Experiments were performed in HEPES (N-2-hydroxyethylpiperazine-N’−2-ethanesulfonic acid)-buffered salt solution supplemented with a physiological concentration of Ca^2+^ (2.5 mM) at room temperature. Cells were stimulated with LPS or A23187 and fluorescence determined every 10 min for 60 min in the CLARIOstar Plus multi-mode microplate reader (BMG Labtech). Excitation and emission wavelengths were 494 and 506 nm, respectively. Cells, not loaded with Fluo-4 AM, were run in parallel and served as blank.

### Agents

LPS (*E. coli* O111:B4, Sigma-Aldrich, cat. no. L2630) was dissolved in PBS. TNF-α and cycloheximide, both from Sigma-Aldrich (cat. no. T7539 and cat. no. C7698), were dissolved in water. A23187 (Sigma-Aldrich, cat. no. C7522) was dissolved in DMSO. Controls received vehicles as appropriate.

### Statistics

Data were analyzed using GraphPad Prism 10 (GraphPad Software). Values are presented as means ± SEM. Each experiment was repeated at least three times. Statistical significance was computed using Student’s two-tailed *t*-test for single comparisons between two groups and one-way ANOVA followed by Sidak’s post-hoc analysis for multiple comparisons as appropriate. P values less than 0.05 were regarded to denote statistical significance.

## Results

### hCASMCs express cytosolic immunoreactivity for MCP-1

**Fig. 1:**
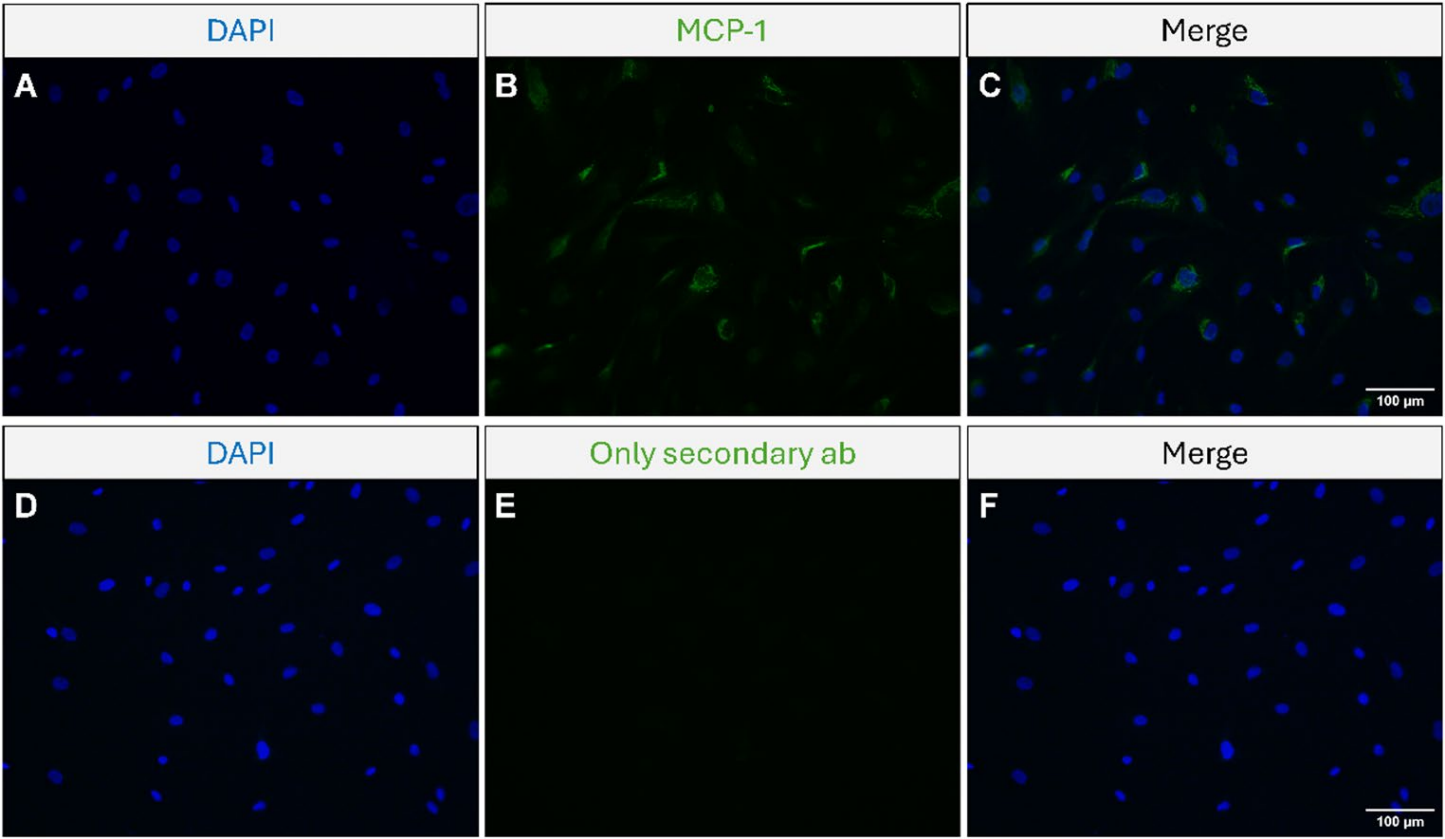
hCASMCs express cytosolic immunoreactivity for MCP-1. **A**–**F** Representative photographs showing the expression of MCP-1 in hCASMCs investigated with immunocytochemistry. The upper row of photographs **A**–**C** shows the same cells stained for nuclei with the nuclear marker DAPI **A**, MCP-1 immunoreactivity **B** and overlay **C**. Omission controls are depicted in the lower row of photographs **D**–**F**. In some cells, the perinuclear region of the cytosol shows rich MCP-1 staining, whereas in other cells immunoreactivity for MCP-1 is scattered in the cytosol and in some cells it is absent. Overlay images of MCP-1 immunoreactivity and DAPI staining were performed in ImageJ Fiji. Bars in panels C and F represent 100 μm for the upper and lower row of photographs, respectively

**Fig. 2:**
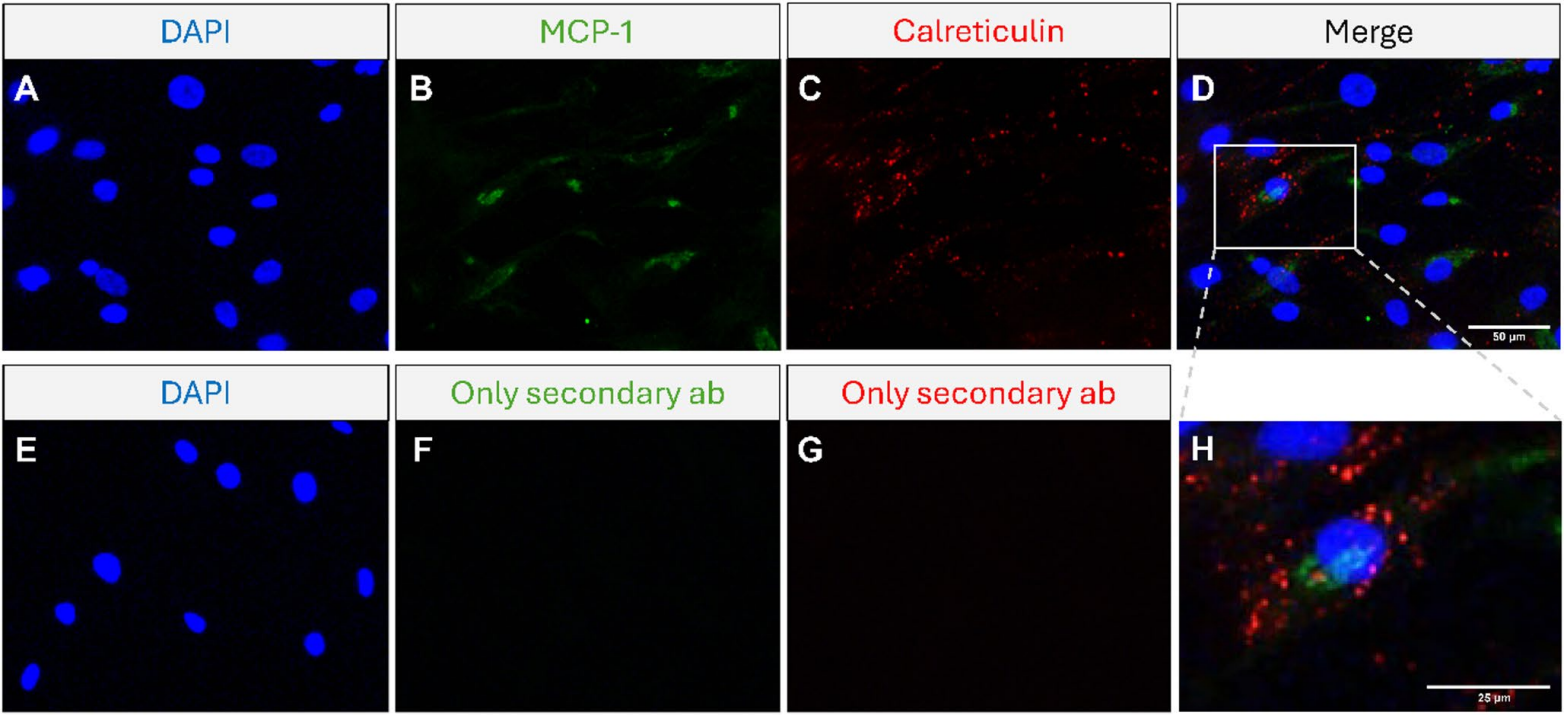
Immunocytochemistry reveals co-staining for MCP-1 and the ER marker calreticulin in hCASMCs. **A**–**H** The same cells were stained with DAPI **A** and incubated with both a mouse monoclonal MCP-1 antibody **B** and a rabbit monoclonal calreticulin antibody **C**. Panel **D** shows overlay and H depicts a zooming in on the area marked in panel **D**. Immunoreactivity for calreticulin was punctuated and detected in the cytosol, and it seemed to co-localize with MCP-1 immunoreactivity **A**–**D**, **H**. No staining was observed after omission of the primary antibodies **E**–**G**. Bar in panel **D** represents 50 μm for panels **A**–**G**

In the first experiments, we assessed expression of MCP-1 in hCASMCs with immunocytochemistry. The hCASMCs showed cytosolic expression of MCP-1 immunoreactivity, whereas no immunoreactive signal for MCP-1 was detected in the nuclei (Fig. 1A–C). MCP-1 immunoreactivity was not detected in all cells (Fig. 1A–C). In some MCP-1 positive cells, the perinuclear region of the cytosol was especially rich in MCP-1 immunoreactivity, whereas in other cells immunoreactivity for MCP-1 was scattered in the cytosol (Fig. 1A–C). The perinuclear MCP-1 immunoreactivity exhibited a punctuated staining pattern (Fig. 1A–C). No immunoreactive signal was observed after omission of the primary MCP-1 antibody included as a negative control (Fig. 1D–F). Next, we performed staining for MCP-1 and the ER marker calreticulin in the same cells (Fig. 2). Immunoreactivity for calreticulin was punctuated and observed in the cytosol as predicted (Fig. 2A, C, D, H). The merging disclosed that MCP-1 and calreticulin seemed to co-stain in the same cell, suggesting that MCP-1 and calreticulin may co-exist (Fig. 2A–D, H). No staining was observed after omission of the primary antibodies (Fig. 2E–G).

### LPS stimulates MCP-1 production in hCASMCs

**Fig. 3:**
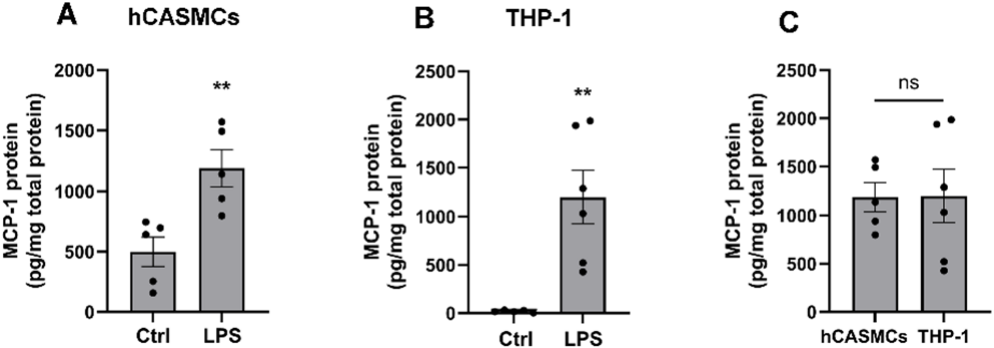
hCASMCs contain high basal levels of MCP-1, and LPS stimulates MCP-1 production in both hCASMCs and THP-1 cells. **A**–**C** hCASMCs and THP-1 monocytes were stimulated with or without LPS (1 µg/ml) for 24 h and MCP-1 levels determined in cell lysates with ELISA. THP-1 monocytes were included as positive control representing an immune cell. MCP-1 amounts were normalized to total protein levels in each sample. Non-stimulated control hCASMCs have higher basal amounts of MCP-1 than THP-1 cells **A**, **B**. LPS increases MCP-1 contents 2–3 times in hCASMCs **A** and by about 60 times in THP-1 cells **B**. hCASMCs and THP-1 cells stimulated with LPS (1 µg/ml) contain similar levels of MCP-1 **C**. Values are presented as means ± SEM. *n* = 5–6 in each group. ** represents *P* < 0.01 vs. control (Ctrl). *ns* not significant. Statistical significance was calculated using Student’s two-tailed *t*-test as appropriate

In the next experiments, we determined basal and LPS-stimulated amounts of MCP-1 in hCASMCs and THP-1 monocyte cell lysates with ELISA and normalized the data to total protein concentration in each sample. THP-1 cells were included as positive control representing an immune cell. The ELISA analysis showed that hCASMCs contained about 25 times higher basal levels of MCP-1 compared to THP-1 cells (Fig. 3A, B). Stimulation with LPS (1 µg/ml) for 24 h increased MCP-1 contents 2–3 times in hCASMCs and by about 60 times in THP-1 cells (Fig. 3A, B). Notably, hCASMCs and THP-1 cells contained similar cellular amounts of MCP-1 after stimulation with LPS (Fig. 3C).

**Fig. 4:**
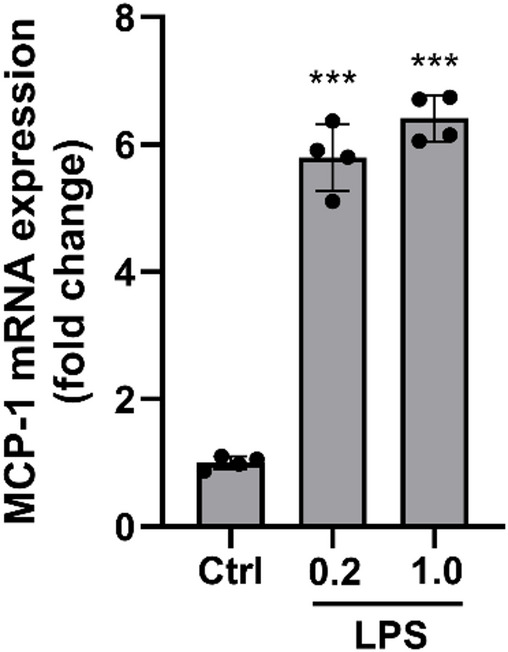
LPS stimulates MCP-1 mRNA expression in hCASMCs. Cells were stimulated with LPS (0.2 and 1 µg/ml) for 24 h and transcript levels for MCP-1 determined with quantitative real-time RT-PCR. Both concentrations of LPS increase the expression of MCP-1 by approximately 6 times. Values are presented as means ± SEM. *n* = 4 in each group. *** represents *P* < 0.001 vs. control (Ctrl). Statistical significance was calculated using one-way ANOVA followed by Sidak’s post-hoc analysis as appropriate

To confirm that LPS enhances production of MCP-1 by hCASMCs, we measured MCP-1 mRNA levels (*CCL2*) in cells stimulated with or without LPS with quantitative real-time RT-PCR. The amounts of *CCL2* transcript were increased by about 6 times in cells stimulated with 0.2–1 µg/ml LPS for 24 h (Fig. 4). Thus, we demonstrate that LPS stimulates both MCP-1 mRNA and protein production.

### Long-term stimulation with pro-inflammatory LPS and TNF-α drives MCP-1 secretion from hCASMCs

**Fig. 5:**
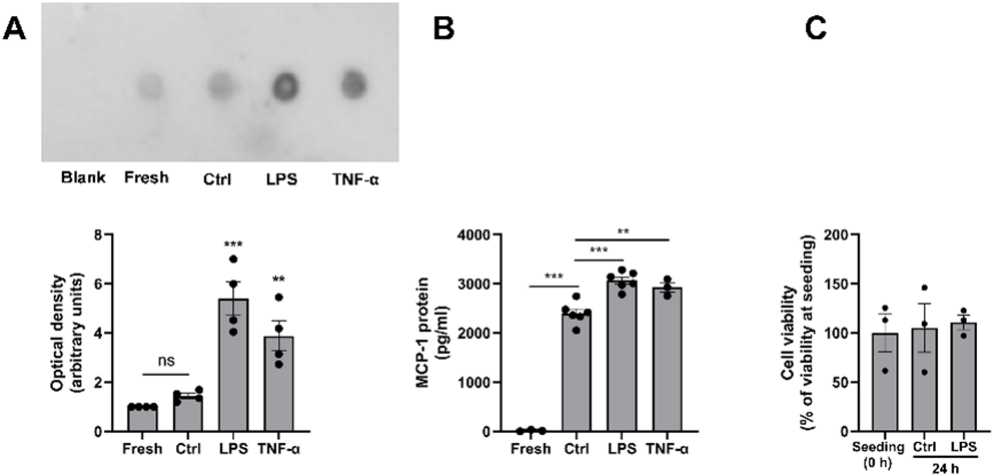
MCP-1 is secreted from hCASMCs in response to pro-inflammatory stimuli. **A**, **B** Conditioned medium was collected from control cells (Ctrl) and cells stimulated with LPS (1 µg/ml) or TNF-α (0.5 ng/ml) for 24 h. MCP-1 contents in fresh and conditioned medium were determined with dot blot **A** and ELISA **B**. Both LPS and TNF-α increase MCP-1 levels in the medium. PBS served as blank. **C** Cell viability at seeding and after treatment with or without LPS determined with the trypan blue exclusion test. After seeding, cells were allowed to equilibrate for 24 h and then treated with or without LPS (1 µg/ml) for 24 h. Values are presented as means ± SEM. *n* = 3–6 in each group. ** and *** represent *P* < 0.01 and *P* < 0.001 vs. Ctrl. ns = not significant. Statistical significance was calculated using one-way ANOVA followed by Sidak’s post-hoc analysis as appropriate

Next, we measured MCP-1 protein levels in culture medium collected from hCASMCs treated with or without LPS using dot blot, to assess if MCP-1 is released from the cells. No difference in MCP-1 immunoreactivity was observed between conditioned medium collected from non-stimulated control cells after 24 h in culture and fresh medium (Fig. 5A). Notably, a background immunoreactive signal for MCP-1 was detected in the fresh culture medium which may mask a difference between fresh and conditioned medium (Fig. 5A). MCP-1 immunoreactivity was elevated by about 4 times in medium from cells stimulated with LPS (1 µg/ml) for 24 h, compared to medium from control cells (Fig. 5A). Medium from cells provoked with another pro-inflammatory stimulus, TNF-α (0.5 ng/ml), expressed about 3 times more MCP-1 immunoreactivity than medium from control cells (Fig. 5A). In the next experiments, we investigated MCP-1 levels with ELISA in medium collected from cells stimulated with LPS and TNF-α to confirm the dot blot data. ELISA analysis showed that conditioned medium collected from non-stimulated control cells contained about 140 times higher levels of MCP-1 compared to fresh medium (Fig. 5B). Stimulation with LPS (1 µg/ml) or TNF-α (0.5 ng/ml) for 24 h further increased MCP-1 contents in the medium by 28 and 22%, respectively, compared to control medium (Fig. 5B). We investigated if cell viability was affected in our experimental settings with the trypan blue exclusion test. In cells treated either with or without LPS (1 µg/ml) for 24 h, cell viability was similar to that at the start of the experiment, showing that LPS has no impact on cell viability (Fig. 5C).

### Short-term stimulation with LPS has no effect on MCP-1 release from hCASMCs

**Fig. 6:**
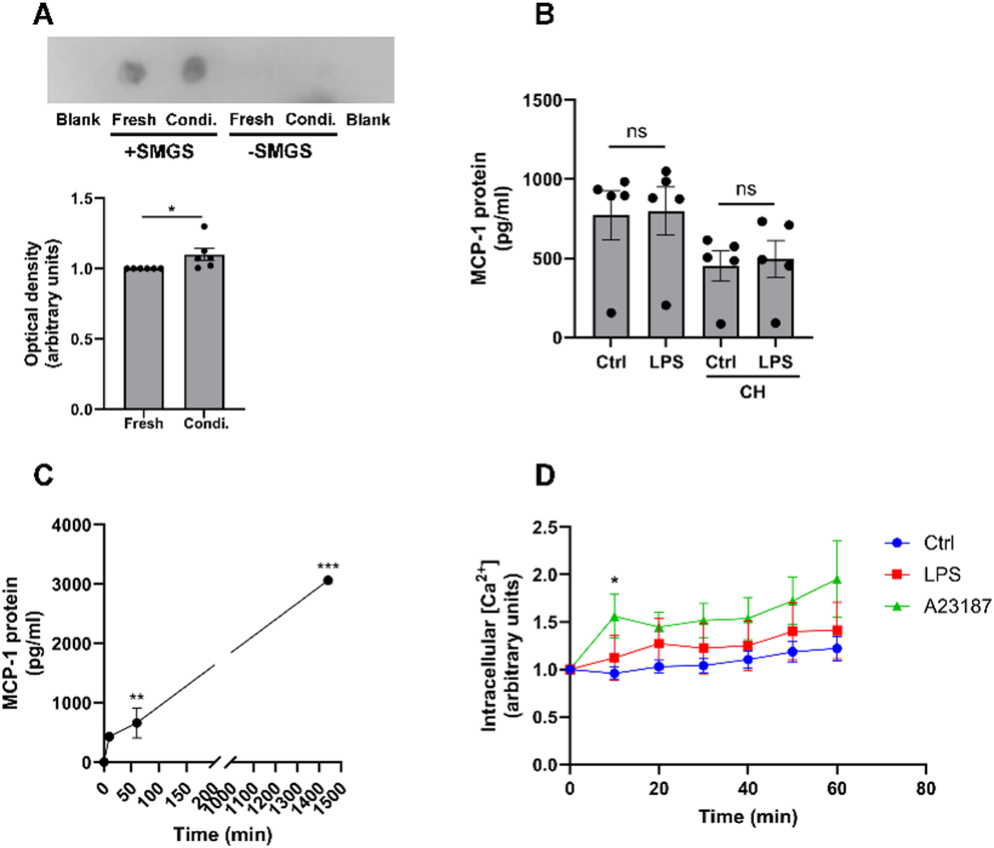
Short-term stimulation with LPS has no effect on MCP-1 secretion from hCASMCs. **A** MCP-1 immunoreactivity is detected in fresh and conditioned (Condi.) medium containing smooth muscle growth supplement (SMGS) but not in medium without SMGS demonstrated with dot blot at a short time point (3 h). Conditioned medium contains more MCP-1 immunoreactivity than fresh medium. PBS was included as blank. **B** hCASMCs were stimulated with or without LPS (1 µg/ml) in the absence or presence of protein synthesis inhibition with cycloheximide (CH, 50 µg/ml) for 60 min and MCP-1 protein levels analyzed in culture medium with ELISA. **C** ELISA analysis shows that MCP-1 levels increase with time in conditioned medium of unstimulated control cells. MCP-1 amounts were assayed at 0-, 10-, 60- and 1440-min. Fresh medium (0 min) contains very low levels of MCP-1. **D** Intracellular [Ca^2+^] was not affected by stimulation with LPS (1 µg/ml), whereas the Ca^2+^ ionophore A23187 (10 µM), included as positive control, increased [Ca^2+^] as predicted. Intracellular [Ca^2+^] was determined with the Ca^2+^-indicator Fluo-4 AM. Values are presented as means ± SEM. *n* = 3–6 in each group. * represents *P* < 0.05 for Ctrl vs. A23187 in panel **D**. ** and *** represent *P* < 0.01 and *P* < 0.001 vs. fresh medium (0 min) in panel C. Ctrl=control. ns = not significant. For data in panels **A** and **B**, statistical significance was calculated with Student’s two-tailed *t*-test and for data in panels **C** and **D** with one-way ANOVA followed by Sidak’s post-hoc analysis as appropriate

Next, we measured MCP-1 release from cells incubated in culture medium supplemented with or without smooth muscle growth supplement at a short time point (3 h) with dot blot. MCP-1 immunoreactivity was detected both in fresh and conditioned medium containing smooth muscle growth supplement but not in medium without growth supplement, suggesting that the background immunoreactive signal is caused by the growth supplement (Fig. 6A). Importantly, conditioned medium collected at 3 h contained more immunoreactivity for MCP-1 than fresh medium (Fig. 6A). To investigate if LPS promotes secretion of stored MCP-1, independent of *de novo* synthesis, we stimulated the hCASMCs with or without LPS (1 µg/ml) for short time (60 min) in the presence or absence of the protein synthesis inhibitor cycloheximide (50 µg/ml) and collected conditioned media for MCP-1 analysis with ELISA. Stimulation with LPS for 60 min had no effect on MCP-1 contents in the culture medium either in the absence or presence of cycloheximide (Fig. 6B). Notably cycloheximide alone reduced secretion of MCP-1, highlighting the importance of protein synthesis for MCP-1 release (Fig. 6B). ELISA analysis revealed that MCP-1 levels in medium of unstimulated control cells increased with time, demonstrating a progressive rise of MCP-1 in the medium (Fig. 6C). In fresh medium (time 0 min), MCP-1 contents were very low, and they increased in conditioned medium already at 10 and 60 min (Fig. 6C). The extracellular concentration of MCP-1 rose by about 165 times to 662 pg/ml already at 60 min (Fig. 6C). This concentration of MCP-1 is similar to that of human plasma and thus relevant for the in vivo situation [9].

Neurotransmitters, hormones and enzyme proteins are stored in secretory vesicles and secretion occurs instantly in response to adequate stimulus, and it is preceded by a rise in intracellular [Ca^2+^] [15–18]. Since LPS has no acute (60 min) stimulatory effect on MCP-1 release from hCASMCs, we investigated the effects of LPS on intracellular [Ca^2+^] within this time frame hypothesizing that LPS lacks effect on intracellular [Ca^2+^]. As predicted, LPS (1 µg/ml) had no effect on intracellular [Ca^2+^], whereas the Ca^2+^ ionophore A23187 (10 µM), included as positive control, caused an increase in intracellular [Ca^2+^] already at 10 min which was maintained over the 60 min observation period (Fig. 6D).

## Discussion

Here, we show that pro-inflammatory stimulus enhances both production and secretion of the important chemokine MCP-1 from hCASMCs, and that hCASMCs contain high basal levels of the chemokine. We reveal using both immunocytochemistry and ELISA, applying different MCP-1 antibodies, that hCASMCs harbour the MCP-1 protein. Interestingly, immunocytochemistry discloses perinuclear staining for MCP-1 in some cells, whereas other cells display MCP-1 immunoreactivity more scattered throughout the cytosol, suggesting that the MCP-1 protein can be redistributed within the cell. Additionally, we observe co-staining for MCP-1 and the ER marker calreticulin, suggesting that they may co-exist. Notably, LPS-induced secretion of MCP-1 from hCASMCs is detected in response to long term (24 h) but not short term (60 min) stimulation with LPS. Furthermore, LPS has no effect on intracellular [Ca^2+^] within 0–60 min, providing additional evidence that LPS has no rapid and direct effect on the MCP-1 secretion. Inhibition of overall protein synthesis with cycloheximide reduces basal MCP-1 release, suggesting that MCP-1 secretion depends on protein synthesis. Thus, our data indicates that LPS-induced release of MCP-1 from hCASMCs is associated with stimulation of MCP-1 protein production, and that it occurs secondary to LPS-induced stimulation of transcription and protein synthesis. LPS triggers production of pro-inflammatory cytokines and chemokines through binding to and activation of its receptor TLR4 leading to down-stream signaling via either MyD88 or TRIF, and activation of NF-κB [19, 20]. Our data suggests that LPS promotes MCP-1 production via this transcriptional signaling pathway causing formation of more secretory vesicles, but we cannot exclude that LPS also acts on later steps in the secretory pathway such as trafficking and fusion of vesicles. Hence, we suggest that synthesis and secretion of MCP-1 show positive correlation.

Interestingly, MCP-1 has been shown to be stored in specific small secretory granules called type-2 chemokine-containing organelle in endothelial cells and secreted through unstimulated exocytosis independent of a preceding rise in intracellular [Ca^2+^] [21, 22]. Hence, there are similarities between hCASMCs and endothelial cells when it comes to secretory mechanisms of MCP-1. Release of MCP-1 has previously been observed and studied in mast cells, but here we for the first time elucidate the mechanisms behind MCP-1 secretion from human vascular smooth muscle cells. Stimulation of MCP-1 production and release is observed in association with mast cell degranulation and is seen simultaneously with secretion of histamine, although degranulation-independent MCP-1 release has also been reported [23, 24]. In primary human mast cells, a spontaneous release of MCP-1 modulated by anti-Ig-E stimulation has been shown [25]. MCP-1 release from mature human mast cells seems to involve a specific type of exocytosis which requires SNAP-23 and syntaxin-3 SNARES [26]. Our data argues that release of MCP-1 is modulated by pro-inflammatory stimulus-induced MCP-1 protein synthesis, and we believe this mechanism to be relevant for both hCASMCs and mast cells. Pharmacological intervention to target MCP-1 secretion may be challenging, because it seems to go hand in hand with protein production. Therefore, approaches directed at MCP-1 gene expression or protein stability may represent more feasible therapeutic strategies.

Our data shows that hCASMCs contain high basal levels of MCP-1, and that they produce and release the chemokine in response to stimulation with pro-inflammatory LPS. LDL, which has been modified by oxidation, has been shown to induce MCP-1 expression in cultured endothelial cells and vascular smooth muscle cells, and to promote migration of monocytes, indicating that oxidized LDL triggers MCP-1 release [3]. Hence, vascular smooth muscle cells, in addition to their classical role in regulation of blood flow and blood pressure, may also act as immune cells through production and release of MCP-1 and thereby recruitment of monocytes to the site of vascular inflammation. Previously, it has been demonstrated that human fibroblasts in the periodontal ligament (PDL fibroblasts) produce cytokines and chemokines, including MCP-1, in response to pro-inflammatory stimulus, besides their traditional part to synthesize collagen, suggesting that PDL cells both build up the extracellular matrix of the PDL tissue and behave as immune cells playing a role in periodontitis [27–30]. One limitation of the present study is that we have not investigated the functional importance of secreted MCP-1 in our experimental settings, e.g. in a transwell migration assay with hCASMCs and monocytes. Another limitation is that we show no data on the expression and localization of MCP-1 in human arteries.

Here, we reveal that hCASMCs contain high amounts of MCP-1. Moreover, we demonstrate for the first time that LPS stimulates secretion of the chemokine from these cells, and that this effect is associated with stimulation of MCP-1 production. LPS-induced production and release of MCP-1 in hCASMCs may represent a mechanism to recruit monocytes to the vascular wall in inflammation.

## Data Availability

All data is included in the article.

## References

[1] Deshmane SL, Kremlev S, Amini S, Sawaya BE (2009) Monocyte chemoattractant protein-1 (MCP-1): an overview. J Interferon Cytokine Res 29:313–326. 10.1089/jir.2008.0027 19441883 10.1089/jir.2008.0027 PMC2755091

[2] Takahashi M, Masuyama J, Ikeda U, Kasahara T, Kitagawa S, Takahashi Y, Shimada K, Kano S (1995) Induction of monocyte chemoattractant protein-1 synthesis in human monocytes during transendothelial migration in vitro. Circ Res 76:750–757. 10.1161/01.res.76.5.750 7728991 10.1161/01.res.76.5.750

[3] Cushing SD, Berliner JA, Valente AJ, Territo MC, Navab M, Parhami F, Gerrity R, Schwartz CJ, Fogelman AM (1990) Minimally modified low density lipoprotein induces monocyte chemotactic protein 1 in human endothelial cells and smooth muscle cells. Proc Natl Acad Sci USA 87:5134–5138. 10.1073/pnas.87.13.5134 1695010 10.1073/pnas.87.13.5134 PMC54276

[4] Anders E, Nebel D, Westman J, Herwald H, Nilsson BO, Svensson D (2018) Globular C1q receptor (p33) binds and stabilizes pro-inflammatory MCP-1: a novel mechanism for regulation of MCP-1 production and function. Biochem J 475:775–786. 10.1042/BCJ20170857 29358188 10.1042/BCJ20170857

[5] Liu L, Bankell E, Rippe C, Morén B, Stenkula KG, Nilsson BO, Swärd K (2021) Cell type dependent suppression of inflammatory mediators by myocardin related transcription factors. Front Physiol 12:732564. 10.3389/fphys.2021.732564 34671275 10.3389/fphys.2021.732564 PMC8521029

[6] Boring L, Gosling J, Cleary M, Charo IF (1998) Decreased lesion formation in CCR2-/- mice reveals a role for chemokines in the initiation of atherosclerosis. Nature 394:894–897. 10.1038/29788 9732872 10.1038/29788

[7] Dawson TC, Kuziel WA, Osahar TA, Maeda N (1999) Absence of CC chemokine receptor-2 reduces atherosclerosis in apolipoprotein E-deficient mice. Atherosclerosis 143:205–211. 10.1016/s0021-9150(98)00318-9 10208497 10.1016/s0021-9150(98)00318-9

[8] Coll B, Alonso-Villaverde C, Joven J (2007) Monocyte chemoattractant protein-1 and atherosclerosis: is there room for an additional biomarker? Clin Chim Acta 383:21–29. 10.1016/j.cca.2007.04.019 17521622 10.1016/j.cca.2007.04.019

[9] de Lemos JA, Morrow DA, Sabatine MS, Murphy SA, Gibson CM, Antman EM, McCabe CH, Cannon CP, Braunwald E (2003) Association between plasma levels of monocyte chemoattractant protein-1 and long-term clinical outcomes in patients with acute coronary syndromes. Circulation 107:690–695. 10.1161/01.cir.0000049742.68848.99 12578870 10.1161/01.cir.0000049742.68848.99

[10] Brenner D, Labreuche J, Touboul PJ, Schmidt-Petersen K, Poirier O, Perret C, Schönfelder J, Combadière C, Lathrop M, Cambien F, Brand-Herrmann SM, Amarenco P, GENIC Investigators (2006) Cytokine polymorphisms associated with carotid intima-media thickness in stroke patients. Stroke 37:1691–1696. 10.1161/01.STR.0000226565.76113.6c 16741188 10.1161/01.STR.0000226565.76113.6c

[11] Libby P (2000) Changing concepts of atherogenesis. J Intern Med 247:349–358. 10.1046/j.1365-2796.2000.00654.x 10762452 10.1046/j.1365-2796.2000.00654.x

[12] Hansson GK, Hermansson A (2011) The immune system in atherosclerosis. Nat Immunol 12:204–212. 10.1038/ni.2001 21321594 10.1038/ni.2001

[13] Libby P, Hansson GK (2015) Inflammation and immunity in diseases of the arterial tree: players and layers. Circ Res 116:307–311. 10.1161/CIRCRESAHA.116.301313 25593275 10.1161/CIRCRESAHA.116.301313 PMC4299915

[14] Pfaffl MW (2001) A new mathematical model for relative quantification in real-time RT-PCR. Nucleic Acids Res 29:e45. 10.1093/nar/29.9.e45 11328886 10.1093/nar/29.9.e45 PMC55695

[15] An D, Lindau M (2024) Exploring the structural dynamics of the vesicle priming machinery. Biochem Soc Trans 52:1715–1725. 10.1042/BST20231333 39082978 10.1042/BST20231333 PMC11357900

[16] Takano T, Yule DI (2024) Neuronal and hormonal control of Ca^2+^ signalling in exocrine glands: insight from in vivo studies. J Physiol 602:3341–3350. 10.1113/JP285461 38847391 10.1113/JP285461 PMC11250672

[17] Sabatini PV, Speckmann T, Lynn FC (2019) Friend and foe: β-cell Ca^2+^ signaling and the development of diabetes. Mol Metab 21:1–12. 10.1016/j.molmet.2018.12.007 30630689 10.1016/j.molmet.2018.12.007 PMC6407368

[18] Park Y, Ryu JK (2018) Models of synaptotagmin-1 to trigger Ca^2+^-dependent vesicle fusion. FEBS Lett 592:3480–3492. 10.1002/1873-3468.13193 30004579 10.1002/1873-3468.13193

[19] Poltorak A, He X, Smirnova I, Liu MY, Van Huffel C, Du X, Birdwell D, Alejos E, Silva M, Galanos C, Freudenberg M, Ricciardi-Castagnoli P, Layton B, Beutler B (1998) Defective LPS signaling in C3H/HeJ and C57BL/10ScCr mice: mutations in Tlr4 gene. Science 282(5396):2085–2088. 10.1126/science.282.5396.2085 9851930 10.1126/science.282.5396.2085

[20] Cheng Z, Taylor B, Ourthiague DR, Hoffmann A (2015) Distinct single-cell signaling characteristics are conferred by the MyD88 and TRIF pathways during TLR4 activation. Sci Signal 8(385):ra69. 10.1126/scisignal.aaa5208 26175492 10.1126/scisignal.aaa5208 PMC6764925

[21] Øynebråten I, Barois N, Hagelsteen K, Johansen FE, Bakke O, Haraldsen G (2005) Characterization of a novel chemokine-containing storage granule in endothelial cells: evidence for preferential exocytosis mediated by protein kinase A and diacylglycerol. J Immunol 175:5358–5369. 10.4049/jimmunol.175.8.5358 16210642 10.4049/jimmunol.175.8.5358

[22] Knipe L, Meli A, Hewlett L, Bierings R, Dempster J, Skehel P, Hannah MJ, Carter T (2010) A revised model for the secretion of tPA and cytokines from cultured endothelial cells. Blood 116:2183–2191. 10.1182/blood-2010-03-276170 20538801 10.1182/blood-2010-03-276170 PMC2951859

[23] Hermans MAW, Schrijver B, van Holten-Neelen CCPA, van Wijk GR, van Hagen PM, van Daele PLA, Dik WA (2018) The JAK1/JAK2- inhibitor ruxolitinib inhibits mast cell degranulation and cytokine release. Clin Exp Allergy 48:1412–1420. 10.1111/cea.13217 29939445 10.1111/cea.13217

[24] Nakayama T, Mutsuga N, Yao L, Tosato G (2006) Prostaglandin E2 promotes degranulation-independent release of MCP-1 from mast cells. J Leukoc Biol 79:95–104. 10.1189/jlb.0405226 16275896 10.1189/jlb.0405226

[25] Tam IYS, Ng CW, Lau HYA, Tam SY (2018) Degradation of monocyte chemoattractant protein-1 by tryptase co-released in immunoglobulin E-dependent activation of primary human cultured mast cells. Int Arch Allergy Immunol 177:199–206. 10.1159/000490533 30021208 10.1159/000490533

[26] Frank SP, Thon KP, Bischoff SC, Lorentz A (2011) SNAP-23 and syntaxin-3 are required for chemokine release by mature human mast cells. Mol Immunol 49:353–358. 10.1016/j.molimm.2011.09.011 21981832 10.1016/j.molimm.2011.09.011

[27] Yamaji Y, Kubota T, Sasaguri K, Sato S, Suzuki Y, Kumada H, Umemoto T (1995) Inflammatory cytokine gene expression in human periodontal ligament fibroblasts stimulated with bacterial lipopolysaccharides. Infect Immun 63:3576–3581. 10.1128/iai.63.9.3576-3581.1995 7642293 10.1128/iai.63.9.3576-3581.1995 PMC173496

[28] Ozaki K, Hanazawa S, Takeshita A, Chen Y, Watanabe A, Nishida K, Miyata Y, Kitano S (1996) Interleukin-1 beta and tumor necrosis factor-alpha stimulate synergistically the expression of monocyte chemoattractant protein-1 in fibroblastic cells derived from human periodontal ligament. Oral Microbiol Immunol 11:109–114. 10.1111/j.1399-302x.1996.tb00344.x 8941762 10.1111/j.1399-302x.1996.tb00344.x

[29] Jönsson D, Nebel D, Bratthall G, Nilsson BO (2011) The human periodontal ligament cell: a fibroblast-like cell acting as an immune cell. J Periodontal Res 46:153–157. 10.1111/j.1600-0765.2010.01331.x 21118418 10.1111/j.1600-0765.2010.01331.x

[30] Nilsson BO (2021) Mechanisms involved in regulation of periodontal ligament cell production of pro-inflammatory cytokines: implications in periodontitis. J Periodontal Res 56:249–255. 10.1111/jre.12823 33305420 10.1111/jre.12823 PMC7984126

